# PSYCHOSOCIAL AND BEHAVIORAL PREDICTORS OF HEALTH AND AGING

**DOI:** 10.1093/geroni/igac059.1464

**Published:** 2022-12-20

**Authors:** Eileen Graham, Nicholas Turiano

## Abstract

Crucial to understanding the aging process is to identify modifiable characteristics that can be targeted in hope of optimizing health across adulthood. This symposium brings together a diverse set of talks about the biopsychosocial and behavioral processes that influence aging outcomes. Turiano used a national sample to demonstrate that individuals with higher levels of neuroticism often use food to cope with stressors which ultimately results in a greater central adiposity and elevated glycated hemoglobin. Zavala used an epidemiological sample of long-banked frozen blood samples to successfully extract DNA and methylation biomarkers from 140 adults and found that stress measures are associated with accelerated biological aging. Luo used coordinated analysis to explore how correlations between personality traits and four health outcomes fluctuate over time, and found the associations between personality (level/change) and health increased in strength through middle adulthood and early stage of late adulthood but weakened in very old age. Jackson found that individuals who experienced high and increasing loneliness in older adulthood had worse cognitive function and steeper cognitive decline than expected given their observed post-mortem neuropathology (i.e., worse cognitive resilience). Lastly, Pfund investigated how personality and social support change together and found within and between person associations between personality and social support, and evidence that retirement was associated with an increase in social support. In sum, this symposium presents novel evidence for the associations among personality, stress, social support, and lonliness are associated with physical and cognitive health in old age.

